# Common Polymorphisms in *MTNR1B*, *G6PC2* and *GCK* Are Associated with Increased Fasting Plasma Glucose and Impaired Beta-Cell Function in Chinese Subjects

**DOI:** 10.1371/journal.pone.0011428

**Published:** 2010-07-08

**Authors:** Claudia Ha Ting Tam, Janice Sin Ka Ho, Ying Wang, Heung Man Lee, Vincent Kwok Lim Lam, Soren Germer, Mitchell Martin, Wing Yee So, Ronald Ching Wan Ma, Juliana Chung Ngor Chan, Maggie Chor Yin Ng

**Affiliations:** 1 Department of Medicine and Therapeutics, The Chinese University of Hong Kong, The Prince of Wales Hospital, Shatin, Hong Kong, Special Administrative Region, People's Republic of China; 2 Li Ka Shing Institute of Health Sciences, The Chinese University of Hong Kong, The Prince of Wales Hospital, Shatin, Hong Kong, Special Administrative Region, People's Republic of China; 3 Hong Kong Institute of Diabetes and Obesity, The Chinese University of Hong Kong, The Prince of Wales Hospital, Shatin, Hong Kong, Special Administrative Region, People's Republic of China; 4 Roche Pharmaceuticals, Nutley, New Jersey, United States of America; University of Bremen, Germany

## Abstract

**Background:**

Previous studies identified melatonin receptor 1B (*MTNR1B*), islet-specific glucose 6 phosphatase catalytic subunit-related protein (*G6PC2*), glucokinase (*GCK*) and glucokinase regulatory protein (*GCKR*) as candidate genes for type 2 diabetes (T2D) acting through elevated fasting plasma glucose (FPG). We examined the associations of the reported common variants of these genes with T2D and glucose homeostasis in three independent Chinese cohorts.

**Methodology/Principal Findings:**

Five single nucleotide polymorphisms (SNPs), *MTNR1B* rs10830963, *G6PC2* rs16856187 and rs478333, *GCK* rs1799884 and *GCKR* rs780094, were genotyped in 1644 controls (583 adults and 1061 adolescents) and 1342 T2D patients. The G-allele of *MTNR1B* rs10830963 and the C-alleles of both *G6PC2* rs16856187 and rs478333 were associated with higher FPG (0.0034<*P*<6.6×10^−5^) in healthy controls. In addition to our previous report for association with FPG, the A-allele of *GCK* rs1799884 was also associated with reduced homeostasis model assessment of beta-cell function (HOMA-B) (*P* = 0.0015). Together with *GCKR* rs780094, the risk alleles of these SNPs exhibited dosage effect in their associations with increased FPG (*P* = 2.9×10^−9^) and reduced HOMA-B (*P* = 1.1×10^−3^). Meta-analyses strongly supported additive effects of *MTNR1B* rs10830963 and *G6PC2* rs16856187 on FPG.

**Conclusions/Significance:**

Common variants of *MTNR1B*, *G6PC2* and *GCK* are associated with elevated FPG and impaired insulin secretion, both individually and jointly, suggesting that these risk alleles may precipitate or perpetuate hyperglycemia in predisposed individuals.

## Introduction

Elevated fasting plasma glucose (FPG) level is an important risk factor contributing to cardiometabolic diseases. Impaired fasting glucose, defined as FPG from 5.6 to 6.9 mmol/l (ADA criteria) [1] or 6.1 to 6.9 mmol/l (WHO criteria) [2], is associated with increased risk of diabetes and cardiovascular disease [3], [4]. Adequate insulin secretion and sensitivity is critical in the maintenance of euglycemia [5]. FPG level is shown to be moderately heritable in twin and family studies (heritability estimate *h^2^* = 0.20–0.28 [6], [7], [8]. Recent genome-wide association studies (GWAS) have uncovered a few loci associated with FPG, including genes encoded for melatonin receptor 1B (MTNR1B), glucose-6-phospate catalytic subunit 2 (G6PC2), glucokinase (GCK) and glucokinase regulatory protein (GCKR) [9]. Surprisingly, these genes only demonstrated modest or weak association with type 2 diabetes (T2D) [9], [10], , despite a close interplay between T2D and high FPG level. Although most T2D genes are implicated in beta-cell function [12], high FPG level from other causes can worsen insulin secretion or sensitivity by setting up a vicious cycle via glucotoxicity [13].

MTNR1B, GCK and G6PC2 proteins are expressed in the human beta cells [14], [15], [16]. *MTNR1B* encodes a high affinity receptor for melatonin, a hormone primarily secreted by the pineal gland to regulate circadian rhythm and sleep cycles [17]. Plasma melatonin follows an opposite circadian rhythm to plasma insulin and glucose, rising by night and falling by day, which suggests that melatonin may affect insulin release and glucose level via its islet-specific receptor [18]. Large scale association studies [9], [10], [14] suggested that two common *MTNR1B* SNPs rs1387153 and rs10830963 (r^2^ = 0.7 in Europeans) may affect insulin secretion and glucose homeostasis.

Glucokinase (encoded by *GCK*) is a glucose-sensing enzyme that determines the threshold for glucose-stimulated-insulin-secretion (GSIS) in islets, and controls gluconeogenesis and glycogen synthesis in hepatocytes [15]. The hepatic activity of GCK is inhibited by its regulatory protein GCKR in a dose-dependent manner, which competes with glucose for binding site [19]. Two *GCKR* variants rs780094 and rs1260326 (r^2^ = 0.93 in Europeans) are associated with HOMA-IR, FPG and triglyceride levels [20], [21]. A functional study showed that this effect was mediated through reduced repression on GCK [22].

The *GCK* variant rs1799884 is associated with higher FPG in Caucasian and Chinese cohorts [23], [24], [25], and rare *GCK* mutations confer a form of maturity-onset diabetes of the young (MODY 2) characterized by hyperglycemia [26]. Glucose-6-phospate catalytic subunit 2 (encoded by *G6PC2*) is proposed to counteract pancreatic GCK activity by competing for glucose usage [16]. G6PC2-null mice demonstrated a ∼15% reduction in FPG level [27], and two SNPs rs560887 and rs563694 were repeatedly associated with hyperglycemia in Europeans [10], [28], [29], [30]. However, these two variants are nearly monomorphic in Asians, so Hu *et al.* adopted a tagSNP approach and reported a FPG-associated SNP rs16856187 in Chinese [31].

Based on their common effects on FPG levels and co-presence in the beta-cell and liver, variants in *MTNR1B*, *GCK*, *G6PC2* and *GCKR* are hypothesized to have interactions or joint effects. Bouatia-Naji *et al.* have reported their additive effects on FPG [9], [32], and we have previously shown that a variant in *GCKR* modifies the association between *GCK* and FPG [24]. In this study, we aimed to validate the reported dosage effects on FPG in a Chinese cohort, and examined their associations with insulin secretion and sensitivity as estimated by HOMA-IR and HOMA-B indices.

## Materials and Methods

### Subjects

We have previously described the study design, ascertainment, inclusion criteria and phenotyping procedures of subjects included in this study [33]. All subjects were of southern Han Chinese ancestry residing in Hong Kong. The control cohort consists of 1644 subjects with FPG<6.1 mmol/l ascertained from a) 583 hospital staff and volunteers from a community-based health screening program (mean age 41.4±10.5 years, 45% male) and b) 1061 adolescents from a community-based school survey (mean age 15.4±1.9 years, 45% male). A subgroup of 420 adult controls also underwent a 75g oral glucose tolerance test (OGTT). The case cohort consists of 1342 unrelated T2D patients (mean age 50.5±13.7 years, 41% male, mean duration of T2D 6.1±6.5 years) selected from the Hong Kong Diabetes Registry (HKDR). T2D was diagnosed according to the 1998 World Health Organization (WHO) criteria. Patients with classic type 1 diabetes with acute ketotic presentation or continuous requirement of insulin within 1 year of diagnosis were excluded. The clinical characteristics of subjects in the three cohorts are summarized in Table 1. Written informed consent was obtained from all adult subjects and parents of the adolescents while the adolescents gave verbal consent. This study was approved by the Clinical Research Ethics Committee of the Chinese University of Hong Kong.

**Table 1:** Clinical and metabolic characteristics of 1644 healthy Chinese adults and adolescents and 1342 T2D patients.

Characteristics	Healthy Adults	Healthy Adolescents	T2D Patients
N (male/female)	583 (265/318)	1061 (481/580)	1342 (544/798)
Age (years)	41.4±10.5	15.4±1.9	50.5±13.7
Age-at-diagnosis (year)	–	–	44.5±13.7
Disease duration (years)	–	–	6.1±6.5
Body mass index (kg/m^2^)	22.9±3.3	19.9±3.5	25.0±4.0
Fasting plasma glucose (mmol/l)	4.8±0.4	4.7±0.3	–
Fasting plasma insulin (pmol/l)	40.8 (25.9–58.6)	45.0 (35.4–60.2)	–

Data are shown as N, mean ± SD or median (interquartile range).

### Clinical studies

All study subjects were examined in the morning after an overnight fast. Anthropometric measurements including body weight and height were documented. Fasting blood samples were collected for DNA extraction and measurements of FPG and fasting plasma insulin (FPI). Homeostasis model assessment of insulin resistance (HOMA-IR) was calculated as (FPI×FPG)
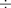
22.5, and homeostasis model assessment of beta-cell function (HOMA-B) was calculated as FPI×20
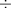
(FPG - 3.5) [34]. Insulinogenic index was calculated as (PI during OGTT for 30 min - 0 min)
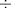
(PG during OGTT for 30 min - 0 min) [35]. Data were discarded if PI or PG level at 0 min were higher than that of 30 min. Insulin sensitivity index (ISI) was estimated using the formula proposed by Matsuda and DeFronzo [36]: 10,000
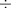
[FPG×FPI×(mean PG during OGTT)×(mean PI during OGTT)]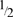. Insulin disposition index (IDI) was calculated as ISI×insulinogenic index
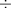
100 [36].

### Genotyping

We genotyped five SNPs in four genes including *MTNR1B* rs10830963, *G6PC2* rs16856187 and rs478333, *GCK* rs1799884 and *GCKR* rs780094 in all study subjects due to their reported associations with FPG, beta-cell function and T2D [29], [31], [32], except for *G6PC2* rs478333. Although *G6PC2* rs560887 [28], [30] and *G6PC2* rs563694 [29] showed association with FPG in Caucasian populations, both of their corresponding minor allele frequencies (MAF) are rare in Chinese population (0.006 and 0.012 in HapMap CHB for rs560887 and rs563694, respectively). Additionally, Shanghai study [31] found that rs16856187 showed the strongest signal for both T2D and FPG. To clarify these inter-ethnic differences, we genotyped rs16856187 and another nearby SNP rs478333 located in the 3′ flanking region, which has common allele frequencies in both Chinese (0.29 in HapMap CHB) and Caucasian (0.49 in HapMap CEU) populations. We did not test for associations for all tagging SNPs of the respective genes. Genotyping on genomic DNA was performed either at deCODE Genetics using the Centaurus (Nanogen) platform or at the McGill University and Genome Quebec Innovation Centre using the Sequenom MassARRAY platform (San Diego, CA, USA). The concordance rate for part of the samples genotyped on both platforms is >99%. All SNPs were in Hardy-Weinberg equilibrium (*P*>0.05) in control cohorts using the exact test implemented in PLINK [37]. The overall genotype call rates were >96% and the minor allele frequencies (MAF) in normal controls (MAF of *MTNR1B* rs10830963 = 0.44 for both adult and adolescent controls; *G6PC2* rs16856187 and rs478333 = 0.30 and 0.35, respectively for both adult and adolescent controls; *GCK* rs1799884 = 0.16 and 0.19 in adult and adolescent controls, respectively) were comparable with the HapMap CHB data (0.48 for *MTNR1B* rs10830963; 0.28 and 0.29 for G6PC2 rs16856187 and rs478333, respectively; 0.20 for *GCK* rs1799884), except the one of *GCKR* rs780094. MAFs of *GCKR* rs780094 in our data (0.46 for both adult and adolescent cohorts) were lower than seen in the HapMap CHB (0.60), but they were similar to the frequency reported in a group of Han Chinese (0.44) study [21].

### Systematic Review

A systematic literature search was performed according to the MOOSE guidelines [38] for the meta-analysis of observational studies. The description of studies and details of the literature search process are outlined in the table (Table S1) and the flow chart (Figure S1), respectively. We searched the PubMed database from inception to January 2010 for association studies between fasting glucose and *MTNR1B* or *G6PC2* genes. The keywords used were MTNR1B, G6PC2 and fasting glucose. We restricted our analysis to human studies, and placed no language restriction. We included studies if they (a) reported the association results for subjects in case-control or population-based studies; (b) genotyped *MTNR1B* rs10830963, *G6PC2* rs560887 or rs16856187 and measured fasting glucose levels for the studied subjects; (c) presented results as mean ± SD with sample size stratified by genotypes with or without adjustment for covariate. We excluded studies if they (a) were reviews or abstract; (b) were duplicate reports on previously published studies; (c) did not provide sufficient information for computation of a quantitative effect estimate of the relationship between FPG and genetic variants.

### Statistical analysis

All data are presented as percentage, mean ± SD or median (interquartile range), as appropriate. Insulinogenic index, FPI, HOMA-IR, HOMA-B, ISI and IDI were logarithmically transformed due to skewed distributions. Each trait was winsorized separately in adult and adolescent cohorts by replacing extreme values with 4 standard deviations from the mean. Less than 0.2% of data were replaced.

Within each control cohort, associations between genotypes and phenotypic traits were tested by multivariate linear regression adjusted for sex, age, and BMI (where appropriate) under the additive genetic model. In the combined analysis, an additional dummy variable “study cohort” coded as 0 for adult controls and 1 for adolescent controls was included in the regression model. Multiple testing of phenotypic traits and SNPs were corrected by controlling the false discovery rate (FDR) using the Benjamini-Hochberg approach [39]. An alternative method for controlling multiple testing is developed by Conneely and Boehnke [40], which accounts for correlation both among SNPs and among phenotypes and is less conservative than FDR. To assess gene-gene interaction effects on phenotypic traits, linear regression analyses including the main and pairwise interaction effects of SNPs under an additive genetic model were applied. The joint effects of the SNPs was assessed by calculating the estimated marginal mean with 95% confidence intervals (CIs) in a general linear model (including sex, age, BMI and study cohort as covariates), categorized by the number of risk alleles assuming an additive genetic model. Risk alleles were defined as alleles that increased fasting plasma glucose described either in literature or in the present study. The significance of the trend was tested by linear regression using the number of risk alleles carried as an independent variable.

Frequencies of genotypes and number of risk alleles between T2D cases and healthy controls were compared using logistic regression adjusted for age, sex and BMI. Odd ratios (ORs) with 95% CIs were presented.

Meta-analyses for the association of FPG were calculated based on the Hedges g statistic which was used to calculate the standardized mean difference (SMD) across studies under the fixed effects model. To account for heterogeneity of SMDs across studies (Cochran's Q statistic *P*<0.1), the overall effect size (SMD) under the random effects model was reported, in which both random variations within and between different studies were incorporated [41].

We estimated study power using genetic power calculator [42]. Assuming an additive model with the frequencies of 0.41 [43] for the G-allele of *MTNR1B* rs10830963, 0.30 [31] for the C-alleles of *G6PC2* rs16856187, 0.17 [24] for the A-allele of *GCK* rs1799884 and 0.54 [24] for the C-allele *GCKR* rs780094 in a Chinese population, our sample size has >90% power to detect a T2D risk under the prevalence of 0.1 with an odds ratio of 1.16 [43], 1.19 [31], 1.22 [25] and 1.18 [21] respectively, and a per-allele effect of increasing FPG by >0.068 (total QTL variance = 0.012) and >0.067 (total QTL variance = 0.012) mmol/l [31], [43] for rs10830963 and rs16856187 respectively, at the α level of 0.05.

All statistical analyses were performed using SAS v.9.1 (SAS Institute, Cary, NC, USA) or SPSS for Windows v.15 (SPSS, Chicago, IL, USA) unless specified otherwise. Two-tailed *P* values <0.05 were considered statistically significant.

## Results

### Associations with FPG, beta cell function and T2D

We have previously shown that the minor A-allele of *GCK* rs1799884 was associated with higher FPG, nevertheless, no association was detected for *GCKR* rs780049. In this study, we further observed consistent and significant association of the minor G-allele of *MTNR1B* rs10830963 (Beta±S.E. = 0.037±0.012 and *P* = 0.0034 in combined analysis) as well as the C-alleles of both *G6PC2* rs16856187 (Beta±S.E. = 0.059±0.015 and *P* = 6.6×10^−5^ in combined analysis) and rs478333 (Beta±S.E. = 0.050±0.013 and *P* = 0.0002 in combined analysis) with increased FPG after adjustment for age, gender, BMI and/or study cohorts (Table 2 and 3).

**Table 2:** Associations of *MTNR1B* rs10830963 with type 2 diabetes related traits in Chinese control subjects.

Study	Genotypes	*n*	BMI (kg/m^2^)	FPG (mmol/l)	FPI (pmol/l)	HOMA-IR	HOMA-B
Adults	CC	181	23±3.4	4.79±0.42	36.0 (23.2–55.9)	1.3 (0.9–2.0)	96.7 (61.6–161.7)
	CG	286	23±3.2	4.84±0.41	44.8 (29.9–60.9)	1.6 (1.0–2.2)	111.8 (75.8–170.9)
	GG	116	22.3±3.2	4.91±0.42	37.3 (24.1–55.8)	1.4 (0.9–2.0)	88.0 (56.8–130.3)
	***P***		0.1268	0.0069	0.2271	0.1234	0.8931
Adolescents	CC	342	19.9±3.5	4.70±0.35	47.8 (35.5–64.1)	1.6 (1.2–2.3)	140.1 (100.4–191.3)
	CG	503	19.8±3.4	4.76±0.33	43.9 (35.0–58.7)	1.6 (1.2–2.1)	124 (94.0–162.6)
	GG	216	20.2±3.7	4.74±0.33	44.9 (36.1–59.5)	1.6 (1.2–2.1)	124.7 (99.1–168.1)
	***P***		0.4961	0.0475	0.2251	0.4303	0.0042
	**Combined ** ***P***		0.8903	0.0034	0.9901	0.6471	0.0532
	**Adjusted ** ***P***		0.9901	0.0162	0.9901	0.8785	0.1685

Data are expressed as N, mean ± SD or median (interquartile range). *P* values were calculated from linear regression adjusted for sex, age and BMI (where appropriate) assuming an additive genetic model. In the combined analysis, calculated *P* values were also adjusted for study cohorts (adult or adolescent). Adjusted *P* values refer to the *P* values controlled for false discovery rate in combined analysis. BMI, body mass index; FPG, fasting plasma glucose; FPI, fasting plasma insulin; HOMA-IR, HOMA of insulin sensitivity; HOMA-B, HOMA of beta- cell function.

**Table 3:** Associations of *G6PC2* rs16856187 and rs478333 with type 2 diabetes related traits in Chinese control subjects.

Study	Genotypes	*n*	BMI (kg/m^2^)	FPG (mmol/l)	FPI (pmol/l)	HOMA-IR	HOMA-B
**rs16856187**							
Adults	AA	262	22.9±3.4	4.80±0.41	41.0 (25.9–56.7)	1.5 (0.9–2.0)	104.4 (70.3–162.2)
	AC	245	22.7±3.0	4.88±0.43	40.5 (26.4–58.9)	1.5 (1.0–2.1)	104.7 (62.9–161.7)
	CC	45	23.6±3.7	4.91±0.35	43.0 (25.9–65.2)	1.5 (0.9–2.4)	110.0 (71.8–184.9)
	***P***		0.9415	0.0428	0.3096	0.2079	0.9689
Adolescents	AA	454	20.0±3.7	4.69±0.33	45.1 (35.0–62.3)	1.6 (1.2–2.2)	133.4 (100.2–178.8)
	AC	414	19.9±3.3	4.77±0.35	44.8 (35.7–59.7)	1.6 (1.2–2.1)	125.5 (92.4–164.2)
	CC	75	19.8±3.6	4.78±0.30	47.5 (35.7–60.0)	1.7 (1.3–2.1)	125.2 (95.1–168.8)
	***P***		0.5112	8.5×10^−5^	0.7023	0.2428	0.0252
	**Combined ** ***P***		0.6473	6.6×10^−5^	0.4416	0.1720	0.1336
	**Adjusted ** ***P***		0.8785	0.0013	0.7997	0.1685	0.3173
**rs478333**							
Adults	TT	238	22.9±3.3	4.79±0.40	41.2 (25.4–60.3)	1.5 (0.9–2.1)	111.3 (67.7–173.7)
	TC	260	22.8±3.1	4.88±0.43	40.0 (25.9–56.8)	1.5 (1.0–2.1)	103.5 (62.5–151.0)
	CC	71	23.0±3.7	4.87±0.42	40.7 (27.2–60.7)	1.5 (0.9–2.2)	100.2 (72.3–160.1)
	***P***		0.9930	0.0561	0.5608	0.4078	0.8290
Adolescents	TT	437	19.9±3.5	4.70±0.33	44.5 (35.2–63.6)	1.5 (1.2–2.2)	134.2 (102.0–182)
	TC	477	20.0±3.4	4.75±0.34	46.3 (35.7–59.5)	1.6 (1.2–2.1)	127.5 (93.9–168.1)
	CC	123	19.8±3.3	4.80±0.33	44.7 (35.2–57.5)	1.6 (1.2–2.0)	114.6 (94.2–158.7)
	***P***		0.9054	0.0008	0.7888	0.7341	0.0068
	**Combined ** ***P***		0.9845	0.0002	0.9287	0.5535	0.0623
	**Adjusted ** ***P***		0.9901	0.0019	0.9901	0.8764	0.1691

Data are expressed as N, mean ± SD or median (interquartile range). *P* values were calculated from linear regression adjusted for sex, age and BMI (where appropriate) assuming an additive genetic model. In the combined analysis, calculated *P* values were also adjusted for study cohorts (adult or adolescent). Adjusted *P* values refer to the *P* values controlled for false discovery rate in combined analysis. BMI, body mass index; FPG, fasting plasma glucose; FPI, fasting plasma insulin; HOMA-IR, HOMA of insulin sensitivity; HOMA-B, HOMA of beta- cell function.

In addition, association with reduced beta-cell function as assessed by HOMA-B was also observed for the A-allele of *GCK* rs1799884 (Beta±S.E. = −0.081±0.026 and *P* = 0.0015), as well as trend for associations with the G-allele of *MTNR1B* rs10830963 (Beta±S.E. = −0.037±0.019 and *P* = 0.0532) and the C-allele of *G6PC2* rs478333 (Beta±S.E. = −0.039±0.021 and *P* = 0.0623) in the combined control samples (Tables 2, 3, 4).

**Table 4:** Associations of *GCK* rs1799884 and *GCKR* rs780094 with type 2 diabetes related traits in Chinese control subjects.

Study	Genotypes	*n*	HOMA-IR	HOMA-B
**GCK rs1799884**				
Adults	GG	399	1.5 (0.9–2.1)	106.4 (66.4–167.8)
	GA	149	1.6 (1.0–2.2)	104.9 (62.8–161.7)
	AA	16	1.4 (0.9–2.4)	102.5 (69.2–132.4)
	***P***		0.6334	0.1551
Adolescents	GG	686	1.6 (1.2–2.2)	133.7 (100.4–178.2)
	GA	303	1.6 (1.2–2.1)	119.5 (90.6–159.4)
	AA	42	1.6 (1.2–2.0)	124.2 (88.0–170.2)
	***P***		0.5414	0.0014
	**Combined ** ***P***		0.8239	0.0015
	**Adjusted ** ***P***		0.9901	0.0095
**GCKR rs780094**				
Adults	TT	123	1.5 (0.9–2.0)	108.9 (67.7–160.1)
	TC	272	1.4 (0.9–2.0)	95.2 (62.2–155.8)
	CC	169	1.5 (1.0–2.4)	112.8 (74.4–183.4)
	***P***		0.3560	0.4322
Adolescents	TT	192	1.6 (1.2–2.1)	128.7 (96.3–181.1)
	TC	441	1.6 (1.2–2.1)	130.1 (97.6–168.2)
	CC	273	1.7 (1.2–2.3)	130.3 (95.1–172.9)
	***P***		0.2892	0.9381
	**Combined ** ***P***		0.1503	0.4630
	**Adjusted ** ***P***		0.3173	0.7997

Data are expressed as N, mean ± SD or median (interquartile range). *P* values were calculated from linear regression adjusted for sex, age and BMI (where appropriate) assuming an additive genetic model. In the combined analysis, calculated *P* values were also adjusted for study cohorts (adult or adolescent). Adjusted *P* values refer to the *P* values controlled for false discovery rate in combined analysis. HOMA-IR, HOMA of insulin sensitivity; HOMA-B, HOMA of beta- cell function.

The associations of FPG with *MTNR1B* rs10830963 (*P* = 0.0162), *G6PC2* rs16856187 (*P* = 0.0013) and rs478333 (*P* = 0.0019), as well as *GCK* rs1799884 with HOMA-B (*P* = 0.0095) in the combined control samples remained significant after controlling for FDR. None of the SNPs revealed association with T2D (Table S2), BMI, FPI or insulin sensitivity, as measured by HOMA-IR (Tables 2, 3, 4) or OGTT-based traits (Table S3).

### Interaction and joint effect of genes on FPG, beta-cell function and T2D

**Figure 1:**
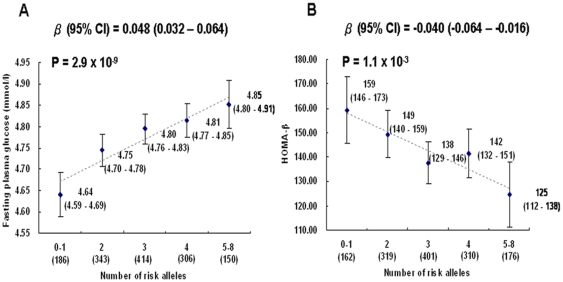
Additive effects on fasting plasma glucose and HOMA beta-cell function. A) Additive effect of the *MTNR1B* rs10830963, G6PC2 rs16856187, *GCK* rs1799884 and *GCKR* rs780094 variants on fasting plasma glucose and B) Additive effect of the *MTNR1B* rs10830963, G6PC2 rs478333, *GCK* rs1799884 and *GCKR* rs780094 variants on HOMA beta-cell function with adjustment for sex, age, BMI and study cohorts (adult or adolescent) in combined control subjects. Values are presented as mean (95% C.I.) according to the number of risk alleles.

We did not detect any novel pairwise interaction between genes on FPG level or beta-cell function in the combined healthy controls (data not shown), apart from the one previously reported between *GCK* rs1799884 and *GCKR* rs780094. We then examined the joint effects of selected SNPs from *GCK*, *GCKR*, *MTNR1B*, *G6PC2* genes on FPG and beta-cell function, assuming that all risk alleles have similar effect sizes. Due to the relatively high linkage disequilibrium between *G6PC2* rs16856187 and *G6PC2* rs478333 (r^2^ = 0.62 in both HapMap CHB data and the present study), only the most significant SNP was included for the joint analysis of FPG or beta-cell function, respectively. We tested for the independence between loci by using all four corresponding loci in the regression models. All loci were independent (*P*<0.05) except for *GCKR* rs780094. Subjects with increasing number of risk alleles showed higher FPG concentration (*P* = 2.9×10^−9^) and lower value of HOMA-B (*P* = 1.1×10^−3^) (Figure 1A–B) in a dose-dependent manner. In addition, we did not find any association between T2D and combined gene variants (Table S2).

### Meta-analyses of MTNR1B rs10830963 and G6PC2 rs16856187 with FPG

In our previous study, we have confirmed the association of *GCK* rs1799884 with FPG in a meta-analysis including both Europeans and Chinese populations. In the present study, meta-analysis of association of *MTNR1B* (rs10830963) in Europeans showed increases of 0.15 (0.11–0.19) and 0.29 (0.25–0.34) in (SMD) of FPG for CG and GG genotypes, respectively, when compared to the CC reference genotype (Figure S2). However, weaker and dominance effect was found in Chinese, with increases of 0.20 (0.11–0.28) and 0.23 (0.12–0.34) in SMD of FPG for CG and GG genotypes, respectively (Figure S2). In the combined meta-analysis for all European and Chinese cohorts, we confirmed the additive effect of the G-allele in *MTNR1B* rs10830963 with FPG. Furthermore, an additive trend of 0.15 (0.03–0.27) and 0.31 (0.19–0.44) increases in SMD of FPG for AC and CC genotypes, respectively, when compared to the AA reference genotype, was also observed for *G6PC2* rs16856187 in Chinese populations (Figure S3). Due to significant heterogeneity amongst the study cohorts (*P*<0.1), the combined SMDs were calculated based on the random effect models, only using the fixed effect models for the association of *MTNR1B* rs10830963 in all Chinese cohorts (*P*>0.1) (Figure S3).

## Discussion

Here we reported the association of rs10830963 in *MTNR1B*, rs16856187 and rs478333 in *G6PC2*, and rs1799884 in *GCK* with higher FPG and lower HOMA-B levels. Further analyses of the risk alleles (G-allele of rs10830963, C-alleles of both rs16856187 and rs478333, A-allele of rs1799884 and C-allele of *GCKR* rs780094) confirmed their joint effects on FPG and beta-cell function. These consistent findings from three independent cohorts strongly support the risk effects of these variants on GSIS to predispose hyperglycemia in Chinese.

Consistent with previous studies [9], [10], [28], [30], [32], [44], [45], [46], [47], we observed the individual and joint effects of risk alleles in *MTNR1B*, *G6PC2*, and *GCK* on FPG. Compared to the carriers with 0 or 1 alleles, each additional allele increases mean FPG level by 0.048 (0.032–0.064) mmol/l (Figure 1). This effect size is similar to those observed in Dutch (0.05 (0.04–0.07)) [32], French (0.07 (0.06–0.08) [9], and Japanese (0.055 (0.045–0.065)) [45] populations, despite the use of different risk variants. While *GCK* rs1799884 and *MTNR1B* rs10830963 are strongly associated to FPG in both Caucasian and Asian cohorts [21], [43], [45], [48], we and others have observed an Asian specific *G6PC2* risk variant (rs16856187 in Hu *et al* [31] and the current study, and rs3755157 in Takeuchi *et al* [45], r^2^≥0.9) in addition to the Caucasian reported rs563694 (Figure S4). This suggests that while these loci are reproducibly associated to FPG across populations, as shown in our meta-analyses (Figures S2, S3), replication in multiple ethnicities could help to identify population specific risk variants and filter for causal variants. Since *G6PC2* rs478333 is common in both Asian (0.29 in Hapmap CHB) and Caucasian (0.49 in HapMap CEU), its replication could provide new insight. Together, the Asian-specific rs16856187 and rs478333 and the Caucasian specific rs563694 (r^2^ = 0.02 and 0.03 to rs16856187 and rs47833, respectively) helped refined a 8.8 kb region of interest (Figure S4), which would be useful for future functional studies.

We further observed that these variants exerted individual and combined effects on beta-cell function, as estimated by the homeostasis model (HOMA-B). To our knowledge, this is the first study showing that the same risk alleles can jointly impair insulin secretion and elevate FPG level. Each allele decreases mean HOMA-B level by 0.04%, which may partially explain the concomitant increase of 0.048 mmol/l in mean FPG level (Figure 1). A recent GWAS meta-analysis involving ∼100,000 Europeans demonstrated that the same risk alleles in *MTNR1B*, *G6PC2*, and *GCK* were associated to FPG and HOMA-B at genome-wide significant levels [48]. Most of the 17 FPG-associated loci were also consistently associated to HOMA-B [48]. Our result and other literature support that impaired beta-cell function and hyperglycemia likely share the same underlying pathogenic mechanism.

Although we had sufficient power (>90%) to detect T2D risks with odds ratio ranging from 1.16 to 1.22 [21], [25], [31], [43], we did not detect T2D-associations for these four loci (*P* = 0.47–0.88). Several large-scale studies failed to find T2D-associations as well [28], [29], and even if found, their effects on T2D are shown to be much weaker than their effects on FPG [9], [10], [11], [21], [31], [32], [43]. Dupuis *et al.* estimated that *MTNR1B*, *GCK* and *GCKR* were associated to T2D with modest effect sizes of 1.06–1.09 [48], so our result may be explained by a lack of power due to the small sample size.

In pancreatic islets, glucose is phosphorylated by GCK into glucose-6-phosphate (G6P), committing it for glycolysis and the subsequent glucose-stimulated insulin secretion (GSIS). G6PC2 is hypothesized to counteract this process, which removes the phosphate group and releases glucose from the beta-cell [16]. MTNR1B may down regulate GCK expression and GSIS by lowering intracellular cAMP level [17], [49]. Indeed, *GCK*, *G6PC2* and *MTNR1B* knock out mice demonstrated significantly lower FPG levels [18], [27], [50]. Thus, the coexistences of risk alleles in *MTNR1B*, *G6PC2*, and *GCK* may confer high melatonin level, low intra-islet glucose oxidation, and low GCK activity in carriers, causing decreased insulin secretion and increased FPG level as observed in this study. The low number of overlapping loci between FPG and T2D GWAS studies [48] suggested that genetic variants may disturb beta-cell function and affect physiological fasting glucose levels beneath the pathological thresholds of T2D.

In conclusion, we showed that risk alleles in *GCK*, *GCKR*, *G6PC2*, and *MTNR1B* exert joint effects on FPG and HOMA-B. The Asian-specific risk variants in *G6PC2* may help to fine map the causal region within the gene (or possibly the *G6PC2-ABCB11* region, given the proximity and strong linkage disequilibrium between the two genes). Concordant with previous studies, we assumed each allele contributes equal dosage, despite minor differences among their effect sizes. Some limitations of this study include using adolescents as controls, which may reduce our power as they may develop T2D later in life. This concern is partially alleviated by obtaining similar results compared to the Chinese and Caucasian adult cohorts (Figures S2, S3). Other studies using adolescent cohorts also reported consistent results [44]. Our study could be improved by using directly measured insulin data rather than surrogate measures such as HOMA-B and HOMA-IR, and increasing the sample size of our OGTT-based associations. Our results could be further improved by using the P*_ACT_* method instead of FDR to correct for multiple comparisons, which considered the correlation structure among both SNPs and phenotypes and is less conservative [40]. Future studies in other populations would substantiate our finding.

## Supporting Information

Table S1 Descriptions of studies.
(0.13 MB DOC)

Table S2 Associations of SNPs and risk allele scores with type 2 diabetes.
(0.07 MB DOC)

Table S3 Associations of MTNR1B rs10830963, GCK rs1799884 as well as G6PC2 rs16856187 and rs478333 with OGTT-based traits in combined Chinese control subjects (adults and adolescents).
(0.07 MB DOC)

Figure S1 Flow chart of literature search for studies on the association of fasting glucose with a) *MTNR1B* rs10830963 and b) *G6PC2* rs560887/rs16856187.
(0.16 MB DOC)

Figure S2 Meta-analysis of associations of *MTNR1B* rs10830963 with fasting plasma glucose in European and Chinese populations.
(0.06 MB DOC)

Figure S3 Meta-analysis of associations of *G6PC2* rs560887 and rs16856187 with fasting plasma glucose in European and Chinese populations, respectively.
(0.06 MB DOC)

Figure S4 Linkage disequilibrium for SNPs within the region near *G6PC2* and *ABCB11* at chromosome 2 between 169.47 Mb and 169.49 Mb.
(0.12 MB DOC)
