# Supplementary material for: Common Polymorphisms in MTNR1B, G6PC2 and GCK Are Associated with Increased Fasting Plasma Glucose and Impaired Beta-Cell Function in Chinese Subjects
Source: PLoS One. 2010 Jul 8;5(7):e11428. doi: 10.1371/journal.pone.0011428 (PMC2900202; doi:10.1371/journal.pone.0011428)
Supplement: Table S1 — Descriptions of studies. (0.13 MB DOC) [file pone.0011428.s001.doc]

**Table S1 Descriptions of studies**

| **Study** | **Cohort** | **Design** | **Sample Size** | **Gender (male / female (%))** | **Mean age** | ***MTNR1B* rs10830963 G allele frequency** | ***G6PC2* rs560887 G allele frequency** | ***G6PC2* rs16856187 C allele frequency** | **Adjustment** |
| --- | --- | --- | --- | --- | --- | --- | --- | --- | --- |
| Prokopenko *et al.* 2009 [1] | CoLaus | population based | 5000 | 46 / 54 | 53.2 | 0.32 | 0.72 |  | gender, age |
|  | deCODE | population based | 6240 | 44.4 / 55.6 | 61.4 | 0.27 | 0.70 |  | gender, age |
|  | DGI | case-control | 1455 | 48.5 / 51.5 | 58.7 | 0.31 | 0.70 |  | gender, age, log BMI, clinical site |
|  | Framingham^a^ | population based | 6479 | 46.0 / 54.0 | 46.0 | 0.28 | 0.70 |  | gender specific residuals adjusted for age and age^2^ |
|  | FUSION | case-control | 1233 | 50 / 50 | 60.7 | 0.33 | 0.69 |  | gender, age, age^2^, birth province, study |
|  | NFBC1966 | population based | 4245 | 49.3 / 50.7 | 31.0 | 0.34 | 0.69 |  | gender, 3 PCs based on GW data determining geographical differences |
|  | NTR/NESDA | population based | 3166 | 33.3 / 66.7 | 43.6 | 0.27 | 0.68 |  | gender, age |
|  | Rotterdam | population based | 2058 | 43 / 57 | 64.0 | 0.28 | 0.69 |  | gender, age |
|  | Sardinia | population based | 4108 | 43.8 / 56.2 | 43.6 | 0.20 | 0.63 |  | gender, age, age^2^, BMI |
|  | TwinsUK^b^ | population based | 1828 | 0 / 100 | 50.2 | 0.30 | 0.71 |  | age |
| Reiling *et al.* 2009 [2] | Dutch New Hoorn | population based | 2361 | 46 / 54 | 53 | 0.25 | 0.69 |  | gender, age, BMI |
| Lyssenko *et al.* 2009 [3] | Botnia PPP | population based | 3300 |  | 48.5 | 0.30 |  |  | gender, age, BMI |
|  | Botnia Prospective (Baseline value) | prospective | 2328 |  | 44.9 | 0.29 |  |  | gender, age, BMI |
|  | Helsinki Birth Cohort | population based | 1600 | 43.6 / 56.4 | 61.6 | 0.34 |  |  | gender, age, BMI |
|  | METSIM | population based | 4257 | 0 / 100 | 59.3 | 0.36 |  |  | gender, age, BMI |
| Ronn *et al.* 2009 [4] | Shanghai | case-control | 1105 | 31.6 / 68.4 | 59.4 | 0.41 |  |  | gender, age, BMI |
| Staiger *et al.* 2008 [5] | GERMAN | population based | 1578 | 33.8 / 66.2 | 40 | 0.30 |  |  | gender, age, BMI |
| Bouatia-Naji *et al.* 2008 [6] | DESIR stage 1 controls | population based | 654 | 41 / 59 | 53.4 |  | 0.67 |  | gender, age, BMI |
|  | DESIR stage 2 controls | population based | 3419 | 48 / 52 | 45.5 |  | 0.70 |  | gender, age, BMI |
|  | NFBC86 | prospective | 5073 | 49 / 51 | 16 |  | 0.70 |  | gender, age, BMI |
|  | Obese French children | population based | 861 | 48 / 52 | 11.02 |  | 0.72 |  | gender, age, BMI |
| Demirci *et al.* 2010 [7] | Non-Hispanic Whites^c^ | population based | 622 | 47 / 53 | 52.8 |  | 0.67 |  | gender, age, BMI, smoking |
| Demirci *et al*. 2010 [7] | Hispanic Americans^c^ | population based | 403 | 49 / 51 | 51.1 |  | 0.83 |  | gender, age, BMI, smoking |
| Rose *et al.* 2009 [8] | Inter99 | population based | 4407 | 46.4 / 53.6 | 45 |  | 0.70 |  | gender, age, BMI |
| Hu *et al.* 2009 [9] | Shanghai | case-control | 1800 | 41.3 / 58.7 | 57.4 |  |  | 0.30 | gender, age, BMI |
| Present study | Hong Kong Adults | population based | 583 | 45 / 55 | 41.4 | 0.44 |  | 0.30 | gender, age, BMI |
|  | Hong Kong Adolescents | population based | 1061 | 45 / 55 | 15.4 | 0.44 |  | 0.30 | gender, age, BMI |

**^a^ mean fasting glucose values are reported for proxy rs7936247 (r2 = 0.59 for rs10830963). ^b^ mean fasting glucose values per genotype are estimated for a subset of unrelated individuals only. ^c^ mean fasting glucose was converted from mg/dl to mmol/l by multiplying 0.0555**

**R REFERENCE**

1. Prokopenko I, Langenberg C, Florez JC, Saxena R, Soranzo N, et al. (2009) Variants in MTNR1B influence fasting glucose levels. Nat Genet 41: 77-81.

2. Reiling E, van 't Riet E, Groenewoud MJ, Welschen LM, van Hove EC, et al. (2009) Combined effects of single-nucleotide polymorphisms in GCK, GCKR, G6PC2 and MTNR1B on fasting plasma glucose and type 2 diabetes risk. Diabetologia.

3. Lyssenko V, Nagorny CL, Erdos MR, Wierup N, Jonsson A, et al. (2009) Common variant in MTNR1B associated with increased risk of type 2 diabetes and impaired early insulin secretion. Nat Genet 41: 82-88.

4. Ronn T, Wen J, Yang Z, Lu B, Du Y, et al. (2009) A common variant in MTNR1B, encoding melatonin receptor 1B, is associated with type 2 diabetes and fasting plasma glucose in Han Chinese individuals. Diabetologia 52: 830-833.

5. Staiger H, Machicao F, Schafer SA, Kirchhoff K, Kantartzis K, et al. (2008) Polymorphisms within the novel type 2 diabetes risk locus MTNR1B determine beta-cell function. PLoS One 3: e3962.

6. Bouatia-Naji N, Rocheleau G, Van Lommel L, Lemaire K, Schuit F, et al. (2008) A polymorphism within the G6PC2 gene is associated with fasting plasma glucose levels. Science 320: 1085-1088.

7. Demirci FY, Dressen AS, Hamman RF, Bunker CH, Kammerer CM, et al. Association of a common G6PC2 variant with fasting plasma glucose levels in non-diabetic individuals. Ann Nutr Metab 56: 59-64.

8. Rose CS, Grarup N, Krarup NT, Poulsen P, Wegner L, et al. (2009) A variant in the G6PC2/ABCB11 locus is associated with increased fasting plasma glucose, increased basal hepatic glucose production and increased insulin release after oral and intravenous glucose loads. Diabetologia 52: 2122-2129.

9. Hu C, Zhang R, Wang C, Ma X, Fang Q, et al. (2009) A genetic variant of G6PC2 is associated with type 2 diabetes and fasting plasma glucose level in the Chinese population. Diabetologia 52: 451-456.
