# Supplementary material for: Common Polymorphisms in MTNR1B, G6PC2 and GCK Are Associated with Increased Fasting Plasma Glucose and Impaired Beta-Cell Function in Chinese Subjects
Source: PLoS One. 2010 Jul 8;5(7):e11428. doi: 10.1371/journal.pone.0011428 (PMC2900202; doi:10.1371/journal.pone.0011428)
Supplement: Table S2 — Associations of SNPs and risk allele scores with type 2 diabetes. (0.07 MB DOC) [file pone.0011428.s002.doc]

**Table S2. Associations of** SNPs and risk allele scores with type 2 diabetes.

|  |  |  | **Genotype frequency (*n*)** | | **Type 2 diabetes** | |
| --- | --- | --- | --- | --- | --- | --- |
| **Gene** | **SNP (risk allele)** | **Genotypes / Alleles** | **T2D** | **Control** | **OR (95% CI)** | ***P*** |
| MTNR1B | rs10830963 (G) | CC | 0.33 (448) | 0.32 (523) | 1.01 (0.87 - 1.17) | 0.8810 |
|  |  | CG | 0.47 (633) | 0.48 (789) |  |  |
|  |  | GG | 0.19 (261) | 0.2 (332) |  |  |
| G6PC2 | rs16856187 (C) | AA | 0.51 (637) | 0.48 (716) | 0.95 (0.8 - 1.12) | 0.5130 |
|  |  | AC | 0.39 (481) | 0.44 (659) |  |  |
|  |  | CC | 0.1 (124) | 0.08 (120) |  |  |
|  | rs478333 (C) | TT | 0.43 (559) | 0.42 (675) | 1.03 (0.89 - 1.21) | 0.6760 |
|  |  | TC | 0.44 (574) | 0.46 (737) |  |  |
|  |  | CC | 0.14 (179) | 0.12 (194) |  |  |
| GCK | rs1799884 (A) | GG | 0.69 (913) | 0.68 (1085) | 1.08 (0.88 - 1.31) | 0.4720 |
|  |  | GA | 0.28 (370) | 0.28 (452) |  |  |
|  |  | AA | 0.03 (37) | 0.04 (58) |  |  |
| GCKR | rs780094 (C) | TT | 0.2 (263) | 0.21 (315) | 1.01 (0.87 - 1.18) | 0.8670 |
|  |  | TC | 0.51 (658) | 0.49 (713) |  |  |
|  |  | CC | 0.29 (381) | 0.3 (442) |  |  |
|  |  |  |  |  |  |  |
| Number of risk alleles | | 0 or 1 | 0.14 (172) | 0.13 (186) | 1.00 (0.92 - 1.1) | 0.9290 |
|  | | 2 | 0.26 (308) | 0.25 (343) |  |  |
|  | | 3 | 0.29 (344) | 0.3 (414) |  |  |
|  | | 4 | 0.19 (231) | 0.22 (307) |  |  |
|  | | 5 - 8 | 0.11 (135) | 0.11 (150) |  |  |

Data are expressed as percentage, *n* or mean ± SD. Risk alleles were defined as alleles that increased fasting plasma glucose described either in literature or in the present study. The risk allele score refers to the total number of risk alleles of rs10830963, rs16856187, rs1799884 and rs780094 under additive models. The ORs (95% CIs) and *P* values for type 2 diabetes were calculated using logistic regression adjusted for sex, age and BMI assuming an additive genetic model. OR (95%CI) were reported with respect to the risk allele. The *P* values for age at diagnosis were calculated using linear regression adjusted for sex and BMI assuming an additive genetic model.
