# Supplementary material for: Common Polymorphisms in MTNR1B, G6PC2 and GCK Are Associated with Increased Fasting Plasma Glucose and Impaired Beta-Cell Function in Chinese Subjects
Source: PLoS One. 2010 Jul 8;5(7):e11428. doi: 10.1371/journal.pone.0011428 (PMC2900202; doi:10.1371/journal.pone.0011428)
Supplement: Table S3 — Associations of MTNR1B rs10830963, GCK rs1799884 as well as G6PC2 rs16856187 and rs478333 with OGTT-based traits in combined Chinese control subjects (adults and adolescents). (0.07 MB DOC) [file pone.0011428.s003.doc]

**Table S3. Associations of *MTNR1B* rs10830963, *GCK* rs1799884 as well as *G6PC2* rs16856187 and rs478333 with OGTT-based traits in combined Chinese control subjects (adults and adolescents)**

| **Gene** | **SNP** | **Genotypes** | ***n*** | **Insulinogenic index** | **ISI** | **IDI** |
| --- | --- | --- | --- | --- | --- | --- |
| *MTNR1B* | rs10830963 | CC | 181 | 18.3 (10.2 - 26.2) | 112.5 (73.7 - 160.4) | 18.4 (12.0 - 29.4) |
|  |  | CG | 286 | 14.7 (9.1 - 23.5) | 95.9 (66.7 - 153.7) | 14.5 (9.4 - 24.0) |
|  |  | GG | 116 | 14.2 (8.6 - 21.7) | 110.3 (81.7 - 153.1) | 14.5 (9.7 - 21.8) |
|  |  | ***P*** |  | 0.0623 | 0.1563 | 0.0059 |
| *GCK* | rs1799884 | GG | 399 | 16.5 (10.2 - 25.0) | 106.6 (71.2 - 160.2) | 15.9 (10.9 - 27) |
|  |  | AG | 149 | 12.6 (7.6 - 23.5) | 98.3 (70.2 - 153.7) | 13.7 (9.2 - 26.1) |
|  |  | AA | 16 | 10.5 (7.5 - 19.6) | 122.4 (85.4 - 160.7) | 14.1 (7.7 - 19.1) |
|  |  | ***P*** |  | 0.1778 | 0.6158 | 0.1237 |
| *G6PC2* | rs16856187 | AA | 262 | 15.7 (9.0 – 23.2) | 104.0 (71.7 – 161.7) | 15.1 (10.1 – 26.4) |
|  |  | AC | 245 | 15.0 (9.2 – 24.0) | 104.3 (70.0 – 154.0) | 15.1 (9.8 – 24.8) |
|  |  | CC | 45 | 16.3 (9.5 – 35.0) | 95.1 (63.3 – 151.7) | 16.6 (8.2 – 28.5) |
|  |  | ***P*** |  | 0.875 | 0.561 | 0.795 |
| *G6PC2* | rs478333 | TT | 238 | 15.4 (8.8 – 23.8) | 107.7 (71.0 – 161.9) | 14.7 (10.1 – 27.1) |
|  |  | TC | 260 | 15.7 (9.4 – 24.4) | 104.6 (72.1 – 155.7) | 15.9 (10.7 – 25.7) |
|  |  | CC | 71 | 14.0 (9.5 – 22.7) | 93.8 (64.7 – 153.2) | 15.1 (8.4 – 25.0) |
|  |  | ***P*** |  | 0.935 | 0.334 | 0.449 |
