# Supplementary material for: Common Polymorphisms in MTNR1B, G6PC2 and GCK Are Associated with Increased Fasting Plasma Glucose and Impaired Beta-Cell Function in Chinese Subjects
Source: PLoS One. 2010 Jul 8;5(7):e11428. doi: 10.1371/journal.pone.0011428 (PMC2900202; doi:10.1371/journal.pone.0011428)
Supplement: Figure S1 — Flow chart of literature search for studies on the association of fasting glucose with a) MTNR1B rs10830963 and b) G6PC2 rs560887/rs16856187. (0.16 MB DOC) [file pone.0011428.s004.doc]

**Figure S1 Flow chart of literature search for studies on the association of fasting glucose with a) *MTNR1B* rs10830963 and b) *G6PC2* rs560887 / rs16856187**

**A**

**
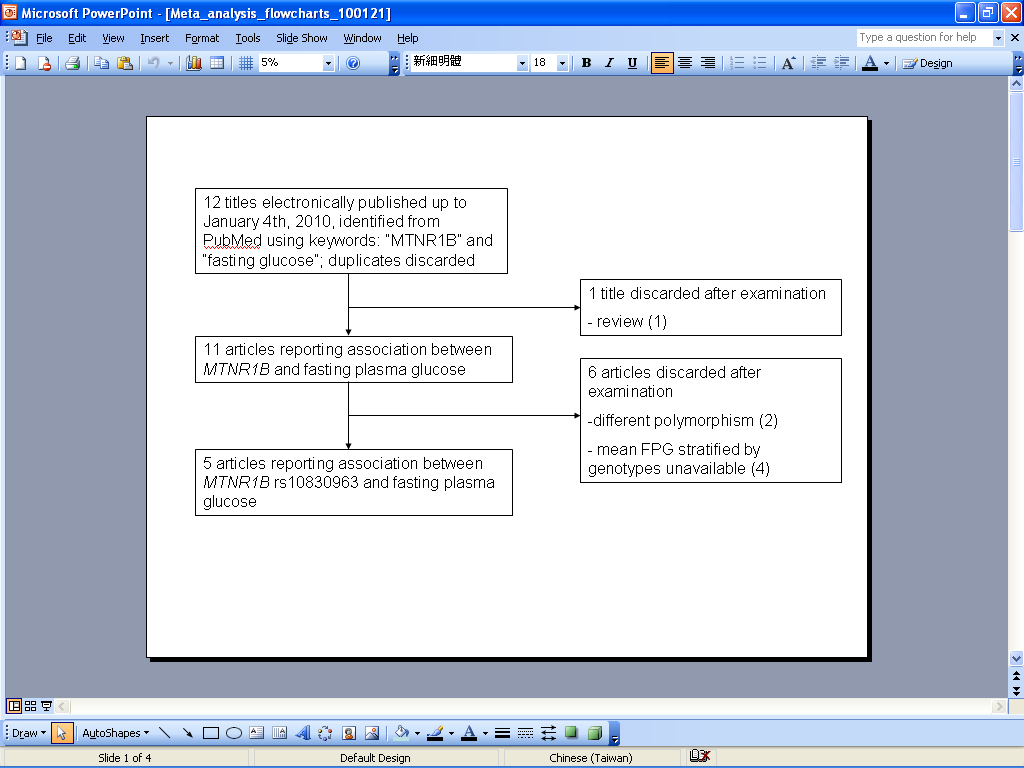
**

**B**

**
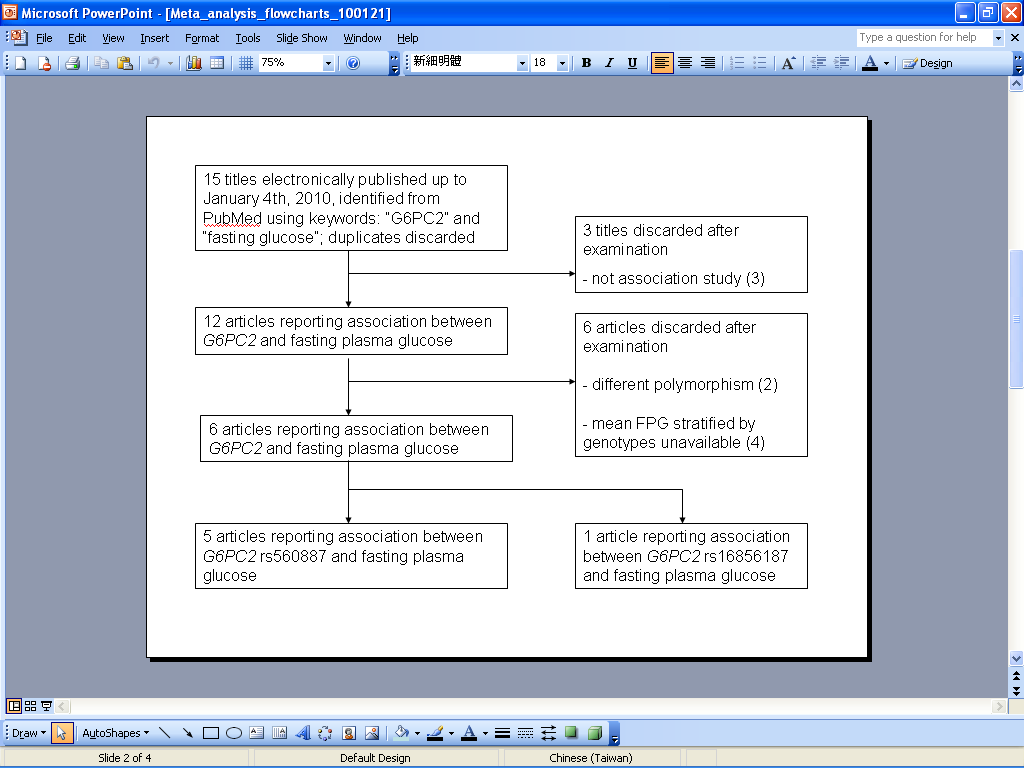
**
