# Supplementary material for: Common Polymorphisms in MTNR1B, G6PC2 and GCK Are Associated with Increased Fasting Plasma Glucose and Impaired Beta-Cell Function in Chinese Subjects
Source: PLoS One. 2010 Jul 8;5(7):e11428. doi: 10.1371/journal.pone.0011428 (PMC2900202; doi:10.1371/journal.pone.0011428)
Supplement: Figure S2 — Meta-analysis of associations of MTNR1B rs10830963 with fasting plasma glucose in European and Chinese populations. (0.06 MB DOC) [file pone.0011428.s005.doc]

**Figure S2 Meta-analysis of associations of *MTNR1B* rs10830963 with fasting plasma glucose in European and Chinese populations. The studies included ten MAGIC studies (CoLaus, deCODE, DGI, Framingham, FUSION, NFBC1966, NTR/NESDA, Rotterdam, Sardinia and TwinsUK) [1], German study [2], four Swedish studies (Botnia PPP, Botnia Prospective (Baseline value), Helsinki Birth Cohort and METSIM) [3], Dutch New Hoorn study [4], Shanghai study [5] and the present study (Chinese adults and adolescents).**

Dutch New Hoorn

**REFERENCE:**

1. Prokopenko I, Langenberg C, Florez JC, Saxena R, Soranzo N, et al. (2009) Variants in MTNR1B influence fasting glucose levels. Nat Genet 41: 77-81.

2. Staiger H, Machicao F, Schafer SA, Kirchhoff K, Kantartzis K, et al. (2008) Polymorphisms within the novel type 2 diabetes risk locus MTNR1B determine beta-cell function. PLoS ONE 3: e3962.

3. Lyssenko V, Nagorny CL, Erdos MR, Wierup N, Jonsson A, et al. (2009) Common variant in MTNR1B associated with increased risk of type 2 diabetes and impaired early insulin secretion. Nat Genet 41: 82-88.

4. Reiling E, van 't Riet E, Groenewoud MJ, Welschen LM, van Hove EC, et al. (2009) Combined effects of single-nucleotide polymorphisms in GCK, GCKR, G6PC2 and MTNR1B on fasting plasma glucose and type 2 diabetes risk. Diabetologia.

5. Ronn T, Wen J, Yang Z, Lu B, Du Y, et al. (2009) A common variant in MTNR1B, encoding melatonin receptor 1B, is associated with type 2 diabetes and fasting plasma glucose in Han Chinese individuals. Diabetologia 52: 830-833.
