# Supplementary material for: Common Polymorphisms in MTNR1B, G6PC2 and GCK Are Associated with Increased Fasting Plasma Glucose and Impaired Beta-Cell Function in Chinese Subjects
Source: PLoS One. 2010 Jul 8;5(7):e11428. doi: 10.1371/journal.pone.0011428 (PMC2900202; doi:10.1371/journal.pone.0011428)
Supplement: Figure S3 — Meta-analysis of associations of G6PC2 rs560887 and rs16856187 with fasting plasma glucose in European and Chinese populations, respectively. (0.06 MB DOC) [file pone.0011428.s006.doc]

**Figure S3 Meta-analysis of associations of *G6PC2* rs560887 and rs16856187 with fasting plasma glucose in European and Chinese populations, respectively. The studies included ten MAGIC studies (CoLaus, deCODE, DGI, Framingham, FUSION, NFBC1966, NTR/NESDA, Rotterdam, Sardinia and TwinsUK) [1], Dutch New Hoorn study [2], four DESIR studies (DESIR stage 1 and 2 controls, NFBC86 and Obese French children) [3], two San Luis Valley Diabetes Studies (Non-Hispanic Whites and Hispanic Americans) [4], Inter99 study [5], Shanghai study [6] and the present study (Chinese adults and adolescents).**

**REFERENCE:**

1. Prokopenko I, Langenberg C, Florez JC, Saxena R, Soranzo N, et al. (2009) Variants in MTNR1B influence fasting glucose levels. Nat Genet 41: 77-81.

2. Reiling E, van 't Riet E, Groenewoud MJ, Welschen LM, van Hove EC, et al. (2009) Combined effects of single-nucleotide polymorphisms in GCK, GCKR, G6PC2 and MTNR1B on fasting plasma glucose and type 2 diabetes risk. Diabetologia.

3. Bouatia-Naji N, Rocheleau G, Van Lommel L, Lemaire K, Schuit F, et al. (2008) A polymorphism within the G6PC2 gene is associated with fasting plasma glucose levels. Science 320: 1085-1088.

4. Demirci FY, Dressen AS, Hamman RF, Bunker CH, Kammerer CM, et al. (2009) Association of a Common G6PC2 Variant with Fasting Plasma Glucose Levels in Non-Diabetic Individuals. Ann Nutr Metab 56: 59-64.

5. Rose CS, Grarup N, Krarup NT, Poulsen P, Wegner L, et al. (2009) A variant in the G6PC2/ABCB11 locus is associated with increased fasting plasma glucose, increased basal hepatic glucose production and increased insulin release after oral and intravenous glucose loads. Diabetologia 52: 2122-2129.

6. Hu C, Zhang R, Wang C, Ma X, Fang Q, et al. (2009) A genetic variant of G6PC2 is associated with type 2 diabetes and fasting plasma glucose level in the Chinese population. Diabetologia 52: 451-456.
