# Supplementary material for: Common Polymorphisms in MTNR1B, G6PC2 and GCK Are Associated with Increased Fasting Plasma Glucose and Impaired Beta-Cell Function in Chinese Subjects
Source: PLoS One. 2010 Jul 8;5(7):e11428. doi: 10.1371/journal.pone.0011428 (PMC2900202; doi:10.1371/journal.pone.0011428)
Supplement: Figure S4 — Linkage disequilibrium for SNPs within the region near G6PC2 and ABCB11 at chromosome 2 between 169.47 Mb and 169.49 Mb. (0.12 MB DOC) [file pone.0011428.s007.doc]

**Figure S4 Linkage disequilibrium for SNPs within the region near *G6PC2* and *ABCB11* at chromosome 2 between 169.47 Mb and 169.49 Mb. Pairwise r^2^ among SNPs for HapMap CEU and CHB are indicated in upper and lower block, respectively. Shades of grey** **represent the strength of pairwise r^2^. “*” refers to SNP genotyped in the present study and “†” refers to SNP showing significant association to fasting plasma glucose in study by Chen *et al.* [1].**

**REFERENCE:**

1. Chen WM, Erdos MR, Jackson AU, Saxena R, Sanna S, et al. (2008) Variations in the G6PC2/ABCB11 genomic region are associated with fasting glucose levels. J Clin Invest 118: 2620-2628.
